# Derivation and Numerical analysis of an Attenuation Operator for non-relativistic waves

**DOI:** 10.1038/s41598-018-34836-3

**Published:** 2018-11-08

**Authors:** Sergio Manzetti

**Affiliations:** 1 Fjordforsk A/S, Bygdavegen 155, 6894 Vangsnes, Norway; 2 Uppsala University, BMC, Dept Mol. Cell Biol, Box 596, SE-75124 Uppsala, Sweden

## Abstract

Quantum mechanical models for particles are strictly dependent on the Schrödinger equation, where the solutions and the Hermitian polynomials form a mathematical foundation to derive expectation values for observables. As for all quantum systems, the solutions are derived in discrete energy levels, and yield probability density, the kinetic energy and average momentum. In this study however, an attenuation Hamiltonian is derived by the algebraic relation of the momentum and position operators, and the derived equation, where the attenuation of kinetic energy is the eigenvalue, is studied numerically. The numerical solutions suggest that the change in kinetic energy from one transition to the next proceeds in an undular fashion, and not in a definite manner. This suggests that any sub-atomic particle which experiences a transition from one level to the next, does so by both gaining and losing energy in an undular manner before reaching an equilibrium with a new and stabilized kinetic energy. The results show also that the phase of the change in kinetic energy between transitions differs between high and low momenta and that higher levels of momentum attenuate more smoothly than transitions between lower energy levels. The investigated attenuation operator may be important for future pinning and quasipinning approaches and play a role in future quantum information processing. Future research is required on the spectrum of the operator and on its potential analytical solutions.

## Introduction

Wavefunction studies are important for the development of various technologies in science, particularly quantum computers, quantum holography systems and laser technologies. Particularly, assigning the values and properties of wavefunctions to algorithms and quantum computational structures is of paramount importance, and several groups have developed various models in the last years^1–3^. Weinacht *et al*. reported a laser-based system where the ionization signals of excited atoms was assigned to specific wavefunction integrals and thus formed the rational structure on the wavefunction-based computational quantum algorithm^1^. Sau *et al*. (2010) recently reported a two-dimensional model and the wavefunction solutions for the *v* = 5/2 fractional quantum Hall system under a high magnetic field as a topological qubit for quantum computational algorithms^2^. Paskaukas and You (2001) showed that the probability density of the wavefunction is a good property to discern between identical particles^3^ and hence can be used to construct wavefunction-sensitive algorithms for quantum computation. These various properties are ultimately applied in the quantum computer which is engineered to form a system of coherent adaptation of the quantum mechanical wavefunction to execute a task^4,5^. For a computational model to make a practical application in a quantum computing system, it must be scalable and hence its solutions (wavefunction, spins, etc.) which are individual qbits, must be represented in a Hilbert space composed of infinitely many dimensions^4^. The wavefunctions can describe electrons, anyons, photons, ions or atoms^5–9^ and can be pinned^10–12^. In order to discern between wavefunctions and particles, operators are paramount. Several groups have developed and studied new quantum operators in recent years, such as non-Hermitian Hamiltonians with special symmetric properties^13^, and complex-scaled Hamiltonians^14^. In this study however, an attenuation Hamiltonian for quantum waves is investigated, where the behaviour of waves between transitions is mapped. Transitions between energy levels can for instance be caused by the release or absorption of a photon, and are given by the form

1 $${\psi }_{nm}(x,t)=a{\psi }_{n}(x,t)+b{\psi }_{m}(x,t)$$

which describes the wavefunction as a mixture of two states, *m* and *n*. The wave-particle entity which is subjected to the transition between the energy levels, *m* and *n*, experiences a change in kinetic energy, which can be described by the form:

2 $${E}_{{\rm{\Delta }}K}={E}_{K,n}-{E}_{K,m},$$

The one-dimensional Schrödinger equation forms the foundation for the derivation of the expectation values of the position, momentum and kinetic energy, which are derive by deploying the algebraic norm on the operators $\hat{x}$, $i\hslash \frac{d}{dx}$ and $-\frac{{\hslash }^{2}}{2m}\frac{{d}^{2}}{d{x}^{2}}$, which are all included as levels of integration in the Hamiltonian kinetic operator:

3 $$H=-\,\frac{{\hslash }^{2}}{2m}\frac{{d}^{2}}{d{x}^{2}},$$

from the Schrödinger equation *Hψ* = *Eψ*.

The Schrödinger Hamiltonian:

4 $$H=-\,\frac{{\hslash }^{2}}{2m}\frac{{\partial }^{2}}{\partial {x}^{2}}+{x}^{2}$$

derives from the general wave equation, which in turn originates from the algebraic properties of the harmonic representation, where the representation is constructed in terms of the two operators P and Q, which respectively define the momentum and the position. The representation of the Schrödinger model, also known as the oscillator representation with an attractive potential is the norm of the operators defined in the association:

5 $$\rho ({x}_{2})\,:\,={P}^{2}+{Q}^{2},$$

which is diagonalized by the Hermite functions on ${\mathbb{R}}$^15^. Other combinations of the norms are given^15^, however such representation describe repulsive potentials (i.e. −*P*^2^).

In order to develop a model for the attenuation in kinetic energy, *P*^3^, during transitions between states *m* and *n*, we consider electrons entirely as waves which are affected by a nuclear potential-well pull, defined by the classical potential term *Q*^2^. This depicts a quantum-relativistic model of transition dynamics for quantum waves, which is proposed to be described by the representation:

6 $$\rho (x)\,:\,={P}^{3}+{Q}^{2},$$

hence requiring the non-self-adjoint third order differential operator:

7 $${P}^{3}=-\,i{\hslash }^{3}\frac{{d}^{3}}{d{x}^{3}}.$$

which is associated to the fluctuations in energy and the harmonic oscillator well-potential in the following relation:

8 $$(-i{\hslash }^{3}\frac{{d}^{3}}{d{x}^{3}}+\frac{1}{2}{k}_{f}{x}^{2})\psi =|{E}_{{\rm{\Delta }}K}|\psi ,$$

where the absolute value of the change in kinetic energy, *E*_Δ*K*_ is the energy difference between energy levels *m* and *n* and $\frac{1}{2}{k}_{f}$ is the harmonic force-constant. The Hamiltonian for the attenuation of a wave under a well potential in a box is therefore proposed to:

9 $${H}_{sa}=-\,i{\hslash }^{3}\frac{{d}^{3}}{d{x}^{3}}+\frac{1}{2}{k}_{f}{x}^{2}$$

where the notation *H*_*sa*_ stands for *s* ingle wave *a* ttenuation Hamiltonian.

The operator in (9) is remarkably simple and grants a simple Hamiltonian which describes attenuation in quantum physics for systems confined by a well-potential. Eqn. (9) can be expanded with additional terms such as electromagnetic field vector potential and be extended to describe wave behaviour and wave changes under various physical phenomena and hence an additional model of study in addition to statistical physics for wave properties. With this in mind, an algebraic outline of the operator properties is made, and the properties of the solutions are ultimately derived numerically in order to study this Hamiltonian as a potential candidate for pinning or quasi-pinning methods for quantum algorithms and quantum measurements of waves and signals.

## Operator Properties

In order to conceive the properties of the modified Schrödinger equation, we wish to study the attributes and the allowed operations of the inherent Hamiltonian (9) in Hilbert space ${\mathcal H}$. This is done by the following set of propositions and proofs.

PROPOSITION I.

*H*_*sa*_ *is linear*.

*Proof*. For *H*_*sa*_ to be linear, the following relation must be satisfied:

10 $${H}_{sa}(f+g)={H}_{sa}(f)+{H}_{sa}(g)$$

Inserting for *H*_*sa*_ in the left-hand side of the equation, and considering the operator under the Heisenberg picture, with $\hslash =1$ and the potential coefficient set to unity:

11 $$[-i\frac{{d}^{3}}{d{x}^{3}}+{x}^{2}](f+g)=[-i\frac{{d}^{3}}{d{x}^{3}}+{x}^{2}](f)+[-i\frac{{d}^{3}}{d{x}^{3}}+{x}^{2}](g).$$

By the sum rule:

12 $$(f+g)\prime\prime\prime =(f)\prime\prime\prime +(g)\prime\prime\prime $$

it follows that:

13 $$[-i\frac{{d}^{3}}{d{x}^{3}}+{x}^{2}](f+g)=(\,-\,if\prime\prime\prime +{x}^{2}f)+(\,-\,ig\prime\prime\prime +{x}^{2}g),$$

giving

14 $${H}_{sa}(f+g)=-\,i(f\prime\prime\prime +g\prime\prime\prime )+{x}^{2}(f+g).$$

For the right-hand side of the equation, we have:

15 $${H}_{sa}(f)+{H}_{sa}(g)=[-i\frac{{d}^{3}}{d{x}^{3}}+{x}^{2}]\,f+[-i\frac{{d}^{3}}{d{x}^{3}}+{x}^{2}]g,$$

which yields:

16 $${H}_{sa}(f)+{H}_{sa}(g)=-\,if\prime\prime\prime +{x}^{2}f-ig\prime\prime\prime +{x}^{2}g,$$

and

17 $${H}_{sa}(f)+{H}_{sa}(g)=-\,i(f\prime\prime\prime +g\prime\prime\prime )+{x}^{2}(f+g).$$

(14) is equal to (17), hence, *H*_*sa*_ is linear.

PROPOSITION II.

*H*_*sa*_ is symmetric for a set of orthogonal functions in a subdomain $${\mathscr{D}}({H}_{sa})$$ in $$ {\mathcal H} $$: $${\mathscr{D}}({H}_{sa})=\{\psi \in  {\mathcal H} :\langle \psi ,{\psi }^{\ast }\rangle =$$$$0\},{\mathscr{D}}({H}_{sa})\subset  {\mathcal H} $$.

*Proof*.

For *H*_*sa*_ to be symmetric in ${\mathscr{D}}({H}_{sa})$, the given relation follows:

18 $$\langle {H}_{sa}\psi ,{\psi }^{\ast }\rangle =\langle \psi ,{H}_{sa}{\psi }^{\ast }\rangle .$$

With *H*_*sa*_ given explicitly and using the Heisenberg picture, with $\hslash =1$:

19 $$\int [-i\frac{{d}^{3}}{d{x}^{3}}+{x}^{2}]\psi {\psi }^{\ast }dx=\int \psi [-i\frac{{d}^{3}}{d{x}^{3}}+{x}^{2}]{\psi }^{\ast }dx,$$

where the left side of the equal sign is equal to:

20 $$\int [-i\frac{{d}^{3}}{d{x}^{3}}+{x}^{2}]\psi {\psi }^{\ast }dx=-\,i\int \frac{{d}^{3}}{d{x}^{3}}\psi {\psi }^{\ast }dx+\int {x}^{2}\psi {\psi }^{\ast }dx,$$

We consider the two terms on the right hand side as ${\rm{A}}=-\,i\int \frac{{d}^{3}}{d{x}^{3}}\psi {\psi }^{\ast }dx$ and ${\rm{B}}=\int {x}^{2}\psi {\psi }^{\ast }dx$:

Solving for A and its two Hermitian counterparts with integration by parts applied with $-i\frac{{d}^{3}}{d{x}^{3}}\psi =u^{\prime}$ and ψ^*^ = *v*:

21 $$-i\int \frac{{d}^{3}}{d{x}^{3}}\psi {\psi }^{\ast }dx=-\,i\frac{{d}^{2}}{d{x}^{2}}\psi {\psi }^{\ast }+i\int \frac{d}{dx}{\psi }^{\ast }\frac{{d}^{2}}{d{x}^{2}}\psi dx$$

and the equivalent for the right hand side in (19):

22 $$-i\int \frac{{d}^{3}}{d{x}^{3}}{\psi }^{\ast }\psi dx=-\,i\frac{{d}^{2}}{d{x}^{2}}{\psi }^{\ast }\psi +i\int \frac{d}{dx}\psi \frac{{d}^{2}}{d{x}^{2}}{\psi }^{\ast }dx,$$

which yields (19) fully written out:

23 $$-i\frac{{d}^{2}}{d{x}^{2}}\psi {\psi }^{\ast }-\,i\int \frac{d}{dx}{\psi }^{\ast }\frac{{d}^{2}}{d{x}^{2}}\psi dx+i\frac{{d}^{2}}{d{x}^{2}}{\psi }^{\ast }\psi +i\int \frac{d}{dx}\psi \frac{{d}^{2}}{d{x}^{2}}{\psi }^{\ast }dx=0$$

which is valid only for orthogonal functions $\psi \perp {\psi }^{\ast }$. We therefore prove:

24 $$\langle A\psi ,{\psi }^{\ast }\rangle -\langle \psi ,A{\psi }^{\ast }\rangle =0,$$

hence, A is symmetric.

Solving for B in (23):

25 $$\int {x}^{2}\psi {\psi }^{\ast }dx=\int \psi {x}^{2}{\psi }^{\ast }dx.$$

which is also valid only for orthogonal functions $\psi \perp {\psi }^{\ast }$. We therefore prove

26 $$\langle B\psi ,{\psi }^{\ast }\rangle -\langle \psi ,B{\psi }^{\ast }\rangle =0,$$

hence B is symmetric. With (*A* + *B*) ⊆ *H*_*sa*_, *H*_*sa*_ is symmetric.

PROPOSITION III.

*H*_*sa*_ is an unbounded operator in ${\mathcal H}$. *H*_*sa*_ is continuous in the compact interval ${\mathscr{D}}({H}_{sa})\subset  {\mathcal H}$, where ${\mathscr{D}}({H}_{sa})\cap {L}^{2}[0,2\pi ]$, and ${L}^{2}\mathrm{[0},2\pi ]\subset {L}^{2}[\,-\,\infty ,+\,\infty ]$.

*Proof*. A proof for unbounded operators is generally related to the unmet criteria for boundedness, where, for *H*_*sa*_ = (*A* + *B*) to be bounded, the following relation must be satisfied:

27 $$||(A)\psi ||\leqslant c||\psi ||$$

28 $$||(B)\psi ||\leqslant c||\psi ||$$

Applying (A) on the given function, *ψ* which is square integrable in *L*^2^[0,2*π*], infinitely differentiable and is thus element of ${\mathcal H}$:

29 $$\psi =\{\begin{array}{lll}cos(n\pi x) & if & 0\leqslant x\leqslant 2\pi /n,\\ 0 & if & 2\pi /n\leqslant x\leqslant 2\pi ,\end{array}$$

gives with $\parallel iA\psi \parallel =|i|\parallel A\psi \parallel =\parallel A\psi \parallel \leqslant c\parallel \psi \parallel$. Using the Heisenberg picture, with $\hslash =1$:

30 $$||{H}_{sa}\psi ||=\sqrt{{\int }_{0}^{2\pi /n}{(-\frac{{d}^{3}}{d{x}^{3}}cos(n\pi x)dx)}^{2}}=\underline{\underline{\sqrt{4{\pi }^{2}{n}^{2}si{n}^{2}\mathrm{(2}{\pi }^{2})}=6.12n}}$$

and,

31 $$c\parallel \psi \parallel =\sqrt{c{\int }_{0}^{2\pi /n}{(cos(n\pi x))}^{2}dx}=\sqrt{c}\sqrt{\pi +sin\mathrm{(4}{\pi }^{2})/(4\pi )}=\underline{\underline{\sqrt{c}1.79}},$$

hence,

32 $$\frac{\parallel {H}_{sa}\psi \parallel }{\parallel \psi \parallel }=\sqrt{c}=\sqrt{3.41}n > n,$$

$A=-\,iA=-\,i\frac{{d}^{3}}{d{x}^{3}}$ does not satisfy condition (25) and (26) and is therefore unbounded.

Applying (28) on (29), the same accounts for B:

33 $$||(B)\psi ||\geqslant c||\psi ||,$$

hence B is unbounded. *H*_*sa*_ = *A* + *B*, whence *H*_*sa*_ is an unbounded operator.

PROPOSITION IV.

*H*_*sa*_ is variant under time-reversal and spatial reflections.

*Proof*.

For *H*_*sa*_ to variant, then the following condition applies:

34 $$\langle \psi ,H\psi \rangle \ne \langle {H}^{\ast }\psi ,\psi \rangle $$

with

35 $${H}_{sa}=-\,i{\hslash }^{3}\frac{{d}^{3}}{d{x}^{3}}+{x}^{2}$$

and

36 $${H}_{sa}^{\ast }=i{\hslash }^{3}\frac{{d}^{3}}{d{x}^{3}}-{x}^{2},$$

we use A and B as above, where ${\rm{A}}=-\,i{\hslash }^{3}\frac{{d}^{3}}{d{x}^{3}}$, ${A}^{\ast }=i{\hslash }^{3}\frac{{d}^{3}}{d{x}^{3}}$, B = *x*^2^ and ${B}^{\ast }=-\,{x}^{2}$. The potential term is treated as a Hermitian function, and by following the Hermitian relationship: *f*(*x*)^*^ = *f*(−*x*), the minus term appears in (36). We use as before, the Heisenberg picture with integration by parts as in (21):

37 $$\langle \psi ,A\psi \rangle =-\,i\frac{{d}^{2}}{d{x}^{2}}{\psi }^{2}+i\int \frac{d}{dx}\psi \frac{{d}^{3}}{d{x}^{3}}\psi dx$$

and

38 $$\langle {A}^{\ast }\psi ,\psi \rangle =i\frac{{d}^{2}}{d{x}^{2}}{\psi }^{2}-\,i\int \frac{d}{dx}\psi \frac{{d}^{3}}{d{x}^{3}}\psi dx,$$

and for B:

39 $$\langle \psi ,B\psi \rangle =\int \psi {x}^{2}\psi dx$$

40 $$\langle \psi ,{B}^{\ast }\psi \rangle =-\,\int \psi {x}^{2}\psi dx\mathrm{.}$$

With 〈*ψ*, *Aψ*〉 ≠ 〈A^*^ψ, *ψ*〉 and 〈*ψ*, *Bψ*〉 ≠ 〈B^*^ψ, *ψ*〉, we have that

41 $$\langle \psi ,H\psi \rangle \ne \langle {H}^{\ast }\psi ,\psi \rangle .$$

*H*_*sa*_ is variant under spatial and temporal transformations. As we consider a mono-dimensional attenuation model in (9), the variance is not expected to change the outcome as the absolute value of the change in energy is used (given in (8)).

PROPOSITION V.

*H*_*sa*_ satisfies the Born-Sommerfeld condition in ${\mathscr{D}}({H}_{sa})\subset  {\mathcal H}$ where ${\mathscr{D}}({H}_{sa})\supseteq Y,{Y}^{\perp }$ where {*ψ*_1_, *ψ*_2_, ..., *ψ*_*n*_} ∈ *Y* and $\{{\psi }_{1}^{\ast },{\psi }_{2}^{\ast }\mathrm{,...,}{\psi }_{n}^{\ast }\}\in {Y}^{\perp }$.

*Proof*.

For an operator to satisfy the Born-Sommerfeld condition, it must be Hermitian and symmetric, which is proved in proposition II. *H*_*sa*_ is therefore Hermitian and probability is conserved. By this, the domain of orthogonal functions ${\mathscr{D}}({H}_{sa})\supseteq Y,{Y}^{\perp }$ is maximal, and hence no boundary conditions have been neglected for the physical problem described by (8).

PROPOSITION VI.

*H*_*sa*_ is a positive operator.

*Proof* Any positive operator has a positive inner-product, 〈*H*_*sa*_*ψ*, ψ^*^〉. Using the infinetly differentiable and square integrable trial function $\psi ={e}^{{x}^{2}}$, we get:

42 $$\langle {H}_{sa}\psi ,\psi \rangle ={\int }_{0}^{2\pi }(-i\frac{{d}^{3}}{d{x}^{3}}+{x}^{2}){e}^{{x}^{2}}{e}^{+{x}^{2}}dx=\frac{8{\pi }^{3}}{3},$$

hence *H*_*sa*_ is a positive operator yielding positive expectation values, when acting on a Hermitian pair of infinitely differentiable functions, similar to the standard Schrödinger Hamiltonian.

### Spectral properties of *H*_*sa*_

#### *Spectrum of H*_*sa*_

For *H*_*sa*_ to have a real spectrum, it must be closed in its domain. We have seen from the previous section that *H*_*sa*_ is an unbounded operator, and hence it’s domain cannot be defined everywhere in ${\mathcal H}$. However, *H*_*sa*_ can be defined in a subdomain domain ${\mathscr{D}}({H}_{sa})$ which is composed of two sets of elements, *Y*_1_ and *Y*_2_, where ${Y}_{1}:\{{\psi }_{11},{\psi }_{12}\mathrm{,...}{\psi }_{1n}\}\perp {Y}_{2}:\{{\psi }_{21},{\psi }_{22},\mathrm{...}{\psi }_{2n}\}$, hence $\{{Y}_{1},{Y}_{2}\}\in {\mathscr{D}}({H}_{sa})$ and ${Y}_{1}\perp {Y}_{2}$.

For *H*_*sa*_ to be closed, the following must be satisfied:

43 $${\psi }_{n}\to \psi \,[{\psi }_{n}\in {\mathscr{D}}({H}_{sa})]$$

and

44 $${H}_{sa}{\psi }_{n}\to \psi ,$$

which is met only for the case of Hermitian sets *Y*_1_ and *Y*_2_. *H*_*sa*_ is therefore closed in a subset $\{{Y}_{1}\perp {Y}_{2}\}\in {\mathscr{D}}({H}_{sa})\subset  {\mathcal H}$, and thus *H*_*sa*_ has a real and non-negative spectrum (see proof VI above) within the sub-domain ${\mathscr{D}}({H}_{sa})$. This set of orthogonal functions forms a closed domain ${\mathscr{D}}({H}_{sa})$, where *H*_*sa*_ is compact. This domain is is the cone in space of principal eigenvalues of the operator and form the spectrum of real averages derived from the solutions of equation (8), via the inner product defined in the closed domain of orthogonal functions (see proof II and V).

If *H*_*sa*_ is not closed on ${\mathcal H}$, then its set of eigenvalues *σ*(*H*_*sa*_) is complex. Inversely, as *H*_*sa*_ is closed on ${\mathcal H}$, its set of eigenvalues is real and the derivation of averages yields real expectation values. *H*_*sa*_ has therefore a complex point spectrum σp(Hsa) outside the sub-domain ${\mathscr{D}}({H}_{sa})$ and a real point spectrum within the sub-domain ${\mathscr{D}}({H}_{sa})$. As stated above, the sub-domain ${\mathscr{D}}({H}_{sa})$ on ${\mathcal H}$ is composed of orthogonal eigenfunctions. In other words, *H*_*sa*_ is an operator that can be defined in various quantum mechanical integrals (i.e. inner product for expectation values or the quantum Lagrangian for exchange statistics) for orthogonal solutions.

#### *H*_*sa*_*is a spectral operator*

*H*_*sa*_ is, as most unbounded operators, a *spectral operator*, a definition which attributes to it special properties, with emphasis on the following:

45 $$({H}_{sa}-\lambda I){\psi }_{n}\to \mathrm{0,}$$

which implies that its continuity has a limit from the left. This gives the particular advantage in that it can be represented as a Riemann-Stieltjes integral, or it can be written by Stone’s theorem^16^ in the form (following the Heisenberg picture):

46 $$\psi (t):={e}^{-t(-i\frac{{d}^{3}}{d{x}^{3}}+{x}^{2})}|{\psi }_{0}(t)\rangle ,$$

which makes the time-dimension, which is strongly continuous, adaptive to the operator *H*_*sa*_. This allows to solve the equation to obtain the state at any subsequent time. This forms a foundation for a time-dependent/-independent mode of analysis of eigenfunctions of *H*_*sa*_.

Finally, as Eq. (45) is assured, we can further outline by Stone’s theorem that every one-parameter group $\{{U}_{t}\}(\,-\,\infty  < t < \infty )$ of unitary transformations admits the spectral representation

47 $${U}_{t}={\int }_{-\infty }^{\infty }{e}^{i\lambda t}d{E}_{\lambda },$$

where {*E*_*λ*_} is a spectral family such that ${E}_{\lambda \cup \cup }\{{U}_{t}\}$. Hence the solutions of the Hamiltonian *H*_*sa*_ invoked in the modified Schrödinger equation in Eq. (8), are composed of terms with complex exponentials with generalized trigonometric polynomials.

### Relationship of *H*_*sa*_ with other quantum operators

The operator *H*_*sa*_ is considered for commutation with the Schrödinger operator in the harmonic potential picture. To begin with, we can inspect the Schrödinger Hamiltonian and the modified Hamiltonian for commutation:

48 $$[{H}_{sa}H\psi ]-[H{H}_{sa}\psi ]=-i{\hslash }^{3}\frac{{d}^{3}}{d{x}^{3}}\times (-\frac{{\hslash }^{2}}{2m}\frac{{d}^{2}}{d{x}^{2}})\psi +\le (\frac{{\hslash }^{2}}{2m}\frac{{d}^{2}}{d{x}^{2}})\times -\,i{\hslash }^{3}\frac{{d}^{3}}{d{x}^{3}}\psi $$

which gives

49 $$[{H}_{sa}H]-[H{H}_{sa}]=0$$

The same outcome is derived for the full Schrödinger operator in the harmonic potential picture with the operator in Eq. (9). By Eq. (49) it can be derived that the operator in Eq. (7) commutes with the kinetic energy and the momentum of a particle. Hence, the attenuation of a kinetic wave, can be defined simultaneously as the momentum and the kinetic energy of a quantum wave.

## Results

The initial analysis of the modified Schrödinger equation in Eq. (8) is performed in the Heisenberg picture, with $\hslash =1$, and with the value of |*E*_Δ*K*_| set for simplicity to a constant, 1, and the coefficient of the harmonic oscillator potential is equally considered at unity. The differential equation studied numerically is therefore,

50 $$[-i\frac{{d}^{3}}{d{x}^{3}}+{x}^{2}]\psi =\psi ,$$

where *ψ* = *ψ*_1_(*x*) + *iψ*_2_(*x*) contains *ψ*_1_(*x*) and *ψ*_2_(*x*) respectively the imaginary and real part of *ψ*. By (50) we obtain the equation

51 $$-i{\psi \prime\prime\prime }_{1}(x)+{x}^{2}{\psi }_{1}(x)+{\psi \prime\prime\prime }_{2}(x)+i{x}^{2}{\psi }_{2}(x)={\psi }_{1}(x)+i{\psi }_{2}(x)$$

and thus the system of differential equations

52 $$A=\{\begin{array}{rcl}{\psi \prime\prime\prime }_{1} & = & (\,-\,1-\,{x}^{2}){\psi }_{2}\\ {\psi \prime\prime\prime }_{2} & = & \mathrm{(1}-{x}^{2}){\psi }_{1}\mathrm{.}\end{array}$$

which are studied numerically using AsymptoticDSolveValue and NDSolve in Mathematica under specific initial conditions (I.C.). The first set of I.C. yields a wavefunction that starts a unity at the zero-point, which is an initial condition conventionally used for harmonic-oscillator solutions to the Schrödinger equation^17^. The derivative is set to zero, in order to indicate a zeroth-order momentum at the start, similar to s-orbitals. The last term, *y*″(0) is considered over several levels, given that the behaviour of the particle’s kinetic energy in a transition between two states *m* and *n* is not measurable by conventional quantum mechanical operators^7^, and hence we represent it by step-wise incremental kinetic energy values. We consider therefore the simple initial conditions for evaluating the pattern of the change in kinetic energy of the wavefunction with the following I.C.s:

53 $$y\mathrm{(0)}=0\,y^{\prime} (0)=0\,y^{\prime\prime} (0)=1,10,15,20,30,50$$

### Approximated series solutions depending on increment in kinetic energy

The Mathematica code AsymptoticDSolveValue was used to derive approximated solutions in series, for the problem given in (52). The solutions represent the functions that describe the energy transitions between energy levels following initial conditions in (53) with a fixed momentum value of zero, and transitions from a kinetic energy factor of 1, 10, 15 and so forth. These functions are approximated polynomials and have immobile local maxima and minima independent on the initial conditions reflecting kinetic energy change (higher levels of n). The real part of the polynomial solution is given by

54 $$\psi {(x)}_{Re}=-\,\frac{k{x}^{10}}{120960}-\frac{k{x}^{8}}{40320}-\frac{k{x}^{7}}{420}+\frac{k{x}^{5}}{120}+\frac{k{x}^{2}}{2},$$

where k is the level of kinetic energy given in (53), hence k can be 1, 10, 15, …, k. All levels have the same stationary points in the wavefunction, respectively −5.88 ∧ 2.04. The imaginary part is equally given as:

55 $$\psi {(x)}_{Im}=-\,\frac{k{x}^{10}}{120960}-\frac{k{x}^{8}}{40320}-\frac{k{x}^{7}}{420}-\frac{k{x}^{5}}{120}+\frac{k{x}^{2}}{2},$$

with stationary points at 2.60 ∧ 4.67.

**Figure 1:**
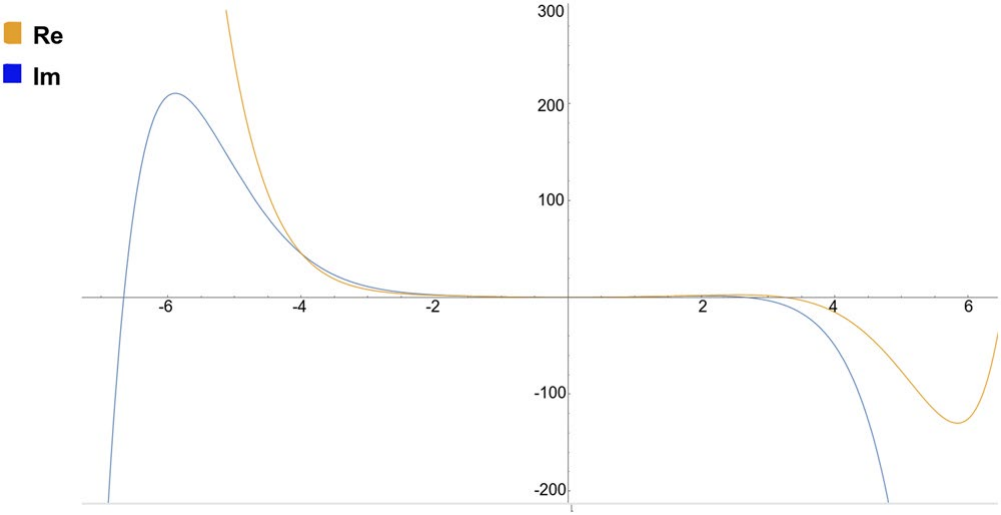
The approximated series solution of the modified Schroedinger equation (52) for the first I.C. given in (53).

The stationary point in Fig. 1 indicate turning points in the plots, where the change in kinetic energy approaches zero. Hence, by following the series solutions given above, one undulation appears to occur in the process of attenuation of kinetic energy for a wave during a transition from level m to n. This suggests that attenuation in kinetic energy does not evolve in a constant fashion during a change from one level to the next, but in an undular manner.

#### Numerical solutions depending on increment in kinetic energy

**Figure 2:**
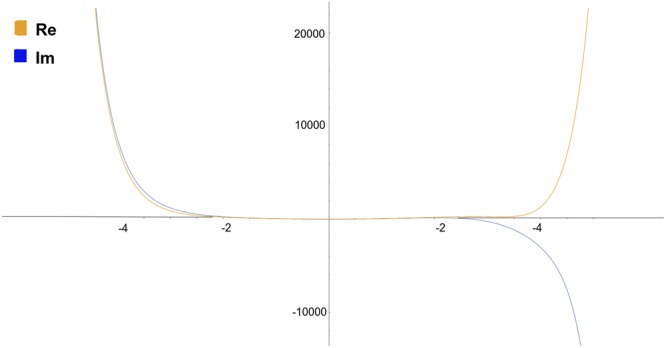
The numerical solution of the modified Schroedinger equation (52) for the initial conditions from Eq. (53). The same plot resulted for all I.C.

The numerical solutions derived using NDSolve depended equally to the series solutions, by the same set of initial conditions in (53) for the problem given in (52). Increments in kinetic energy gave no change in the pattern of the wavefunction (Fig. 2), similar to the series approximations with stationary equilibrium points. This indicates that the process of transition between levels occurs in an equal fashion for any defined interval of potential energy Δ*E*.

#### Numerical solutions depending on energy level transition

**Figure 3:**
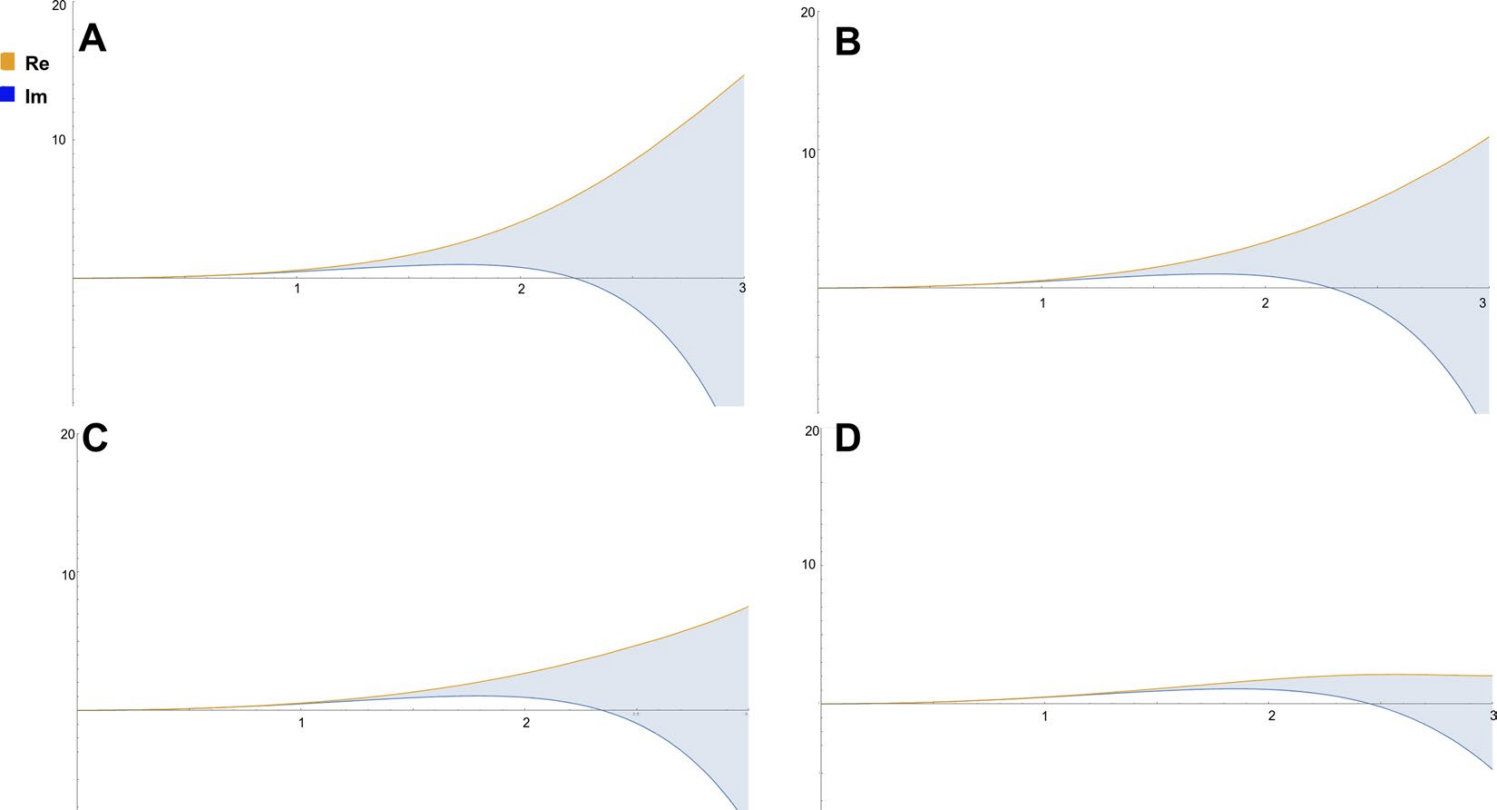
The numerical solutions of the modified Schroedinger equation (52) depending on variations of Δ*E* from A (large value of Δ*E* - quantum level) towards the classical limit, D (small value of Δ*E*).

As a third part of the numerical analysis, it is equally critical to determine the effect of the width of the excitation gap between energy levels on the solutions of Eq. (52). The lowest energy transitions have larger gaps than the transitions which reach the classical limits, hence the behaviour of the solutions to (52) where the energy gap is defined by variable value Δ*E*:

56 $$[-i\frac{{d}^{3}}{d{x}^{3}}+{x}^{2}]\psi ={\rm{\Delta }}E\psi ,$$

is investigated in Mathematica^18^ and shown in Fig. 3, where Δ*E* is modulated from a low value (narrow excitation gap - classical limit) to a high value (wide excitation gap - quantum level).

### Approximated series solutions depending on increment in momentum

In order to study the pattern of energy transition for different start-momenta (i.e s-, p-, d- and f- and g- orbitals), the initial conditions:

57 $$y(0)=0\,y^{\prime} (0)=1,2,3,4,5\,y^{\prime\prime} (0)=1,$$

are used to form series solutions to the problem in Eq. (52) by differences in momentum of the waves. The real components of the series solutions following increment in momentum (subscript) are:

58 $$\begin{array}{rcl}\psi {(x)}_{1} & = & \frac{29{x}^{10}}{3628800}+\frac{{x}^{9}}{15120}-\frac{{x}^{8}}{40320}-\frac{13{x}^{7}}{5040}-\frac{{x}^{6}}{120}+\frac{{x}^{5}}{120}+\frac{{x}^{4}}{24}+\frac{{x}^{2}}{2}+x\\ \psi {(x)}_{2} & = & \frac{{x}^{10}}{129600}+\frac{{x}^{9}}{7560}-\frac{{x}^{8}}{40320}-\frac{{x}^{7}}{360}-\frac{{x}^{6}}{60}+\frac{{x}^{5}}{120}+\frac{{x}^{4}}{12}+\frac{{x}^{2}}{2}+2x\\ \psi {(x)}_{3} & = & -\frac{{x}^{10}}{134400}+\frac{{x}^{9}}{5040}-\frac{{x}^{8}}{40320}-\frac{{x}^{7}}{336}-\frac{{x}^{6}}{40}+\frac{{x}^{5}}{120}+\frac{{x}^{4}}{8}+\frac{{x}^{2}}{2}+3x\\ \psi {(x)}_{4} & = & \frac{13{x}^{10}}{1814400}+\frac{{x}^{9}}{3780}-\frac{{x}^{8}}{20160}-\frac{{x}^{7}}{315}-\frac{{x}^{6}}{30}+\frac{{x}^{5}}{120}+\frac{{x}^{4}}{6}+\frac{{x}^{2}}{2}+4x\\ \psi {(x)}_{5} & = & -\frac{{x}^{10}}{145152}-\frac{{x}^{9}}{3024}-\frac{{x}^{8}}{40320}-\frac{17{x}^{7}}{5040}-\frac{{x}^{6}}{24}+\frac{{x}^{5}}{120}+\frac{5{x}^{4}}{24}+\frac{{x}^{2}}{2}+5x\end{array}$$

The imaginary components of the solutions are given as:

59 $$\begin{array}{rcl}\psi {(x)}_{1} & = & \frac{29{x}^{10}}{3628800}-\frac{{x}^{9}}{15120}-\frac{{x}^{8}}{40320}-\frac{11{x}^{7}}{5040}-\frac{{x}^{6}}{120}-\frac{{x}^{5}}{120}-\frac{{x}^{4}}{24}+\frac{{x}^{2}}{2}+x\\ \psi {(x)}_{2} & = & -\frac{{x}^{10}}{129600}-\frac{{x}^{9}}{7560}-\frac{{x}^{8}}{40320}-\frac{{x}^{7}}{360}-\frac{{x}^{6}}{60}-\frac{{x}^{5}}{120}-\frac{{x}^{4}}{12}+\frac{{x}^{2}}{2}+2x\\ \psi {(x)}_{3} & = & -\frac{{x}^{10}}{134400}-\frac{{x}^{9}}{5040}-\frac{{x}^{8}}{40320}-\frac{{x}^{7}}{336}-\frac{{x}^{6}}{40}-\frac{{x}^{5}}{120}-\frac{{x}^{4}}{8}+\frac{{x}^{2}}{2}+3x\\ \psi {(x)}_{4} & = & -\frac{13{x}^{10}}{1814400}-\frac{{x}^{9}}{3780}-\frac{{x}^{8}}{40320}-\frac{{x}^{7}}{315}-\frac{{x}^{6}}{30}-\frac{{x}^{5}}{120}-\frac{{x}^{4}}{6}+\frac{{x}^{2}}{2}+4x\\ \psi {(x)}_{5} & = & -\frac{{x}^{10}}{145152}-\frac{{x}^{9}}{3024}-\frac{{x}^{8}}{40320}-\frac{17{x}^{7}}{5040}-\frac{{x}^{6}}{24}-\frac{{x}^{5}}{120}-\frac{5{x}^{4}}{24}+\frac{{x}^{2}}{2}+5x\end{array}$$

The real components have the following nodes with the x-axis, shown in Table 1 (where the attenuation of energy is zero - stable states).

**Table 1:** Stationary points of series solutions (zero attenuation).

*Momentum*	*ψ*(0)_1_	*ψ*(0)_2_	*ψ*(0)_3_
1	−0.92	2.45	5.06
2	−14.86	2.41	4.79
3	2.36	5.68	23.20
4	−33.19	2.37	4.61
5	−43.27	−3.49	2.19

**Figure 4:**
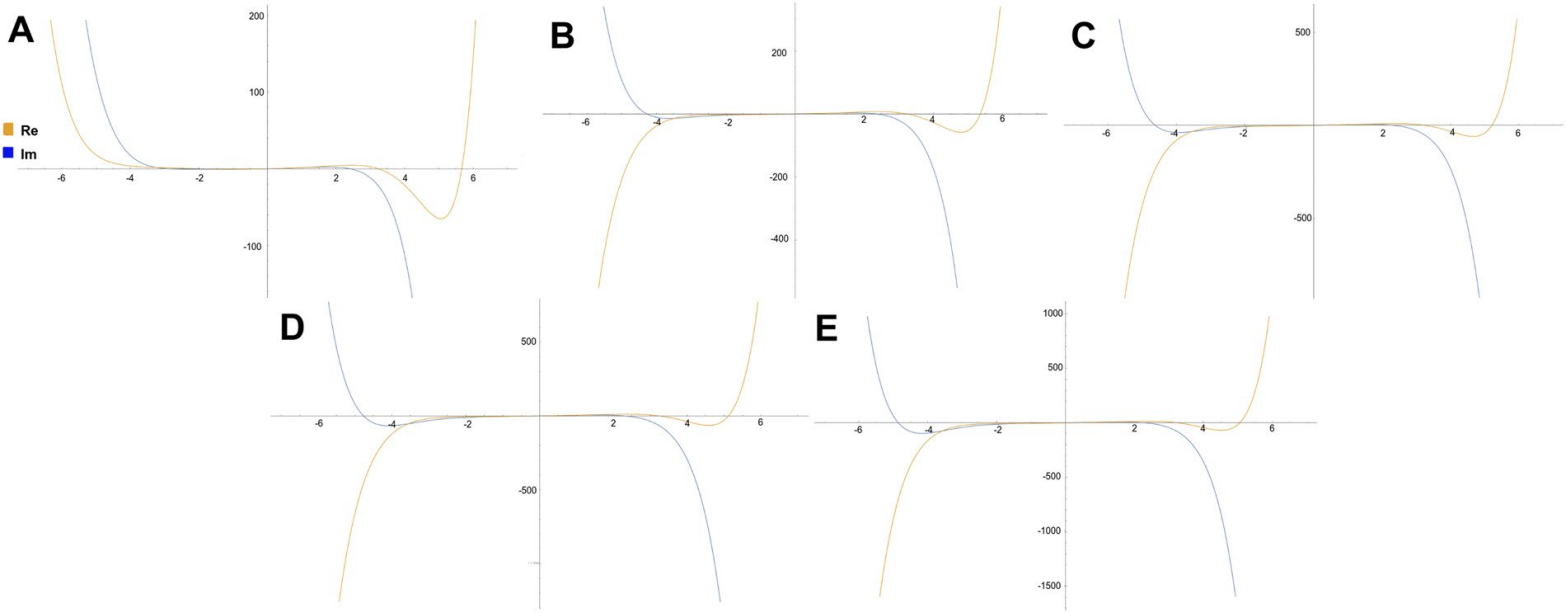
The approximated series solutions of the modified Schroedinger equation (52) by increment in momentum (A-E refers to momenta in Table 1) in the initial conditions.

In an equal fashion, the imaginary components have variable stationary points (data not shown). The variation of stationary points suggests that the attenuation proceeds by different undulation patterns for waves of different momentum. The stationary points are positions where the attenuation is zero, given that the wavefunction is rectilinear. A zero attenuation indicates an intermediate stationary state in change of energy. The plots of the 5 states of Eqs 58 and 59 are shown in Fig. 4.

#### Numerical solutions depending on increment in momentum

**Figure 5:**
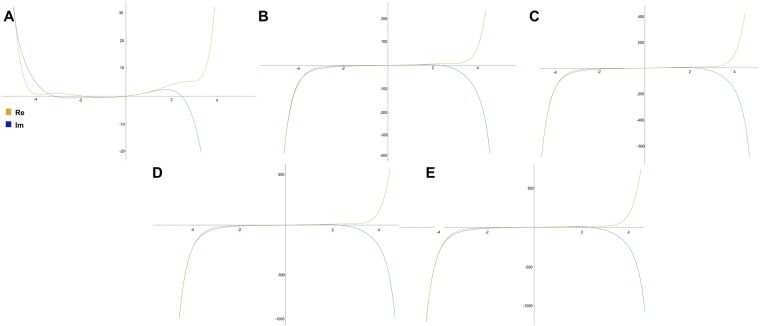
The numerical solutions of the modified Schroedinger equation (52) by increment in momentum (A-E refers to momenta in Table 1) in the initial conditions.

The values of the momentum in the initial conditions are, equally to the previous section, studied numerically. The plots show a trend where an increment in momentum in the initial conditions yields a real part of the solutions that evolves from having an undular behaviour within a well-like parabolic curve towards forming a hyperbolic curve, similar to the hyperbolic trigonometric function sinh(x) (Fig. 5), while the inverse occurs for the imaginary part. Accounting for that the imaginary part is related to the differential operator, and the real part is closely associated to the effect from the potential term *x*^2^, it suggests that zero momentum (i.e. s-orbitals) allows the wave to attenuate energy in a dynamic undular fashion (with several stationary points in attenuation), when absorbing energy and undergoing a transition to the next level of momentum (i.e. s- to p-orbital). Higher transitions (i.e. from p- to d-, or d- to f-) yield instead a smoother attenuation of energy from one level to the next.

## Discussion

The first point to consider for discussion is the resulting existence of stationary points in the attenuation pattern of a wave, when undergoing a transition between energy levels. The first series-wave functions in (54) which can be attributed to describe the attenuation of energy between any level of kinetic energy with fixed momentum (n = 1 → n = 2, n = 1 ← n = 2, n = 40 → n = 41 or n = 40 ← n = 41) show the particular property of having two preserved stationary points occurring for any transition. Noting that the transitions only differ by start-level of kinetic energy, the existence of two stationary points where the attenuation of energy is zero, defines that any transition proceeds in an undular manner, independent of the state. This suggests that the absorption or the release of a photon does not proceed linearly, but harmonically. The implications of this feature are that “quasi-states” can occur between energy levels, and that energy is exchanged bidirectionally, rather than uni-directionally. A second point of interest for discussion from the results are the implications of the momentum of the wave when discerning between transitions. The series solutions in Eq. (58) and also the numerical solutions (Figs 2 and 4), show that attenuation of energy for wave with differing momentum proceed in different patterns. The first point of interest lies in the variation of the distribution of the stationary points in (58). These stationary points, where the attenuation of the wave is zero, appear in narrower intervals for sharp orbitals, slightly wider interval for principal orbitals and wide intervals for diffuse and fundamental orbitals. In other words, attenuation of energy for s-orbitals (i.e. 1 s to 2 s) can be pinned to proceed with a high “frequency” in attenuation of energy. If the orbitals are more evolved in angular momentum (i.e. d orbitals), the attenuation proceeds more smoothly, with wider infinitesimal time-periods between stationary phases of attenuation. A third point of discussion is the property of the third order differential operator, which is a non-self-adjoint operator. Non-self-adjoint operators in quantum mechanics are less common, however some researchers have studied non-self-adjoint Hamiltonians in context with particular physical problems. Hokkyo^19^ studied the role of adjoint wavefunctions for non-self-adjoint Hamiltonians in nuclear reactor models. Hokkyo used the non-self-adjoint representation to study the solutions for unstable states which can be exemplified by non-relativistic mechanisms such as the Gamow theory of *α*-decay and the unstable V particle in the NVfJ model^19^. Davies^20^ indicate that the resolvent norms of non-self-adjoint quantum operators with complex potentials becomes very large even though the complex variable is far from the spectrum of the operator. This may imply the existence of non-trivial or simply incorrect eigenvalues the more $\hslash \to 1$. In this study we have considered the Eq. (8) under the Heisenberg picture, where $\hslash \mathrm{=1}$ for computational limitations, however the operator yields real eigenvalues (see section on Spectrum properties) and should therefore not suffer for complex eigenvalues. However, the operator is not self-adjoint, and has no upper or lower limit, contrary to the regular Schrödinger operator which has a lower limit. This may however not suffer for physical inconsistencies, and rather represent a challenge in determining its spectrum. At last, one wishes to ask the following question: How do these results serve the scientific community? There are two answers to this question. The first is the educational perspective and scientific value of conceiving that all processes of energy-exchange occur most-likely by wave-like patterns. The second answer is the value to technology development. Although infinitesimal time-periods reign the attenuation process of waves, the differentiation of attenuation between waves of unequal momentum may be a relevant property of quantum waves for pinning and quasi-pinning of states in the development.

## Conclusions

A new wave model has been derived for waves affected by a well potential by the algebraic relations of the momentum and position operators. The attenuation Hamiltonian includes the imaginary third order differential operator, which - in similarity to the second order differential operator from the regular Schrödinger equation - has algebraic properties that allow for orthogonal functions to be derived analytically. The attenuation Hamiltonian yields a wave-attenuation model with non-quantized solutions which describe the change of the kinetic energy of waves subjected to a well potential, during transitions from one energy level to the next. This Hamiltonian can describe the release and absorption of photons and be an important operator for pinning and quasi-pinning algorithms.

## Methods

The numerical analysis and derivation of approximated series solutions is conducted using NDSolve and AsymptoticDSolveValue with the Mathematica Development Platform^18^. The  Mathematica protocol is available at Fjordforsk A.S. website, http://www.fjordforsk.no/sci_rep_1860.zip.
